# Cytotoxicity evaluation of curcumin treatment in DH82 canine histiocytic sarcoma cell line

**DOI:** 10.1186/1753-6561-8-S4-P138

**Published:** 2014-10-01

**Authors:** Natália Noronha, Gabriel Silva, Ana Lucia Fachin, Mozart Marins

**Affiliations:** 1 Department of Biotechnology, University of Ribeirão Preto, 14096-900, Ribeirão Preto, São Paulo, Brazil

## Background

The identification of targets and new drugs to fight cancer is one the greatest challenges for Biotechnology researchers. Not only humans, but also animals are affected by this disease. It is estimated for example that one in every 3-4 dogs will develop some type of cancer during its lifetime, twice as much as the human being [1,2]. DNA methylation is an important epigenetic mechanism which control gene expression during cell proliferation and differentiation. Deregulation of this mechanism is one of the events which can contribute to the development of cancer [3]. The identification of epigenetic drugs, which are molecules that can revert aberrant DNA methylation, is one of the main areas of cancer research nowadays. In this regard, natural products are a rich source of possibilities to increase the arsenal of epigenetic drugs [1]. This study aims to analyze the cytotoxicity effect of curcumin treatments in DH82 canine histiocytic sarcoma cell line. This polyphenol derived from *Curcuma longa *has emerged as a potent *multimodal *cancer-preventing agent which modulates multiple cell signaling pathways and also been described as an inhibitor of DNA methylation[4]. The knowledge of the biological effects of these substances can pinpoint targets for the development of epigenetic drugs, with a positive impact for the treatment of canine and human cancer.

## Materials and methods

DH82 cells grew under 37°C and 5% CO_2, _DMEM medium was supplemented with 10% Bovine Fetal Serum. To estimate DH82 viability and curcumin cytotoxicity, the MTT protocol was performed at a concentration of 2 × 10^5 ^cells/well cultured in 96 well plates. After each treatment, the cells were incubated during four hours with a 5 μg/mL MTT (3 - (4,5-dimethylthiazol-2-yl) -2,5-diphenyltetrazolium bromide) solution. Lastly the MTT salt conversion to formazan crystals was quantified by spectrophotometry.

A curcumin therapeutic dose curve was established at 24, 48 and 72 hours treatments.

## Results and discussions

In 24 hours, the IC_50 _was 21.88µg/mL (59.4µM), in 48 hours was 14.26µg/mL (38.7µM) and in 72 hours was 12.17µg/mL (33.0µM). A range of values were obtained and the dispersion, especially on IC_50 _coordinate coincides with the trend line, showing good positive correlation.

## Conclusions

The DH82 cell line demonstrated susceptibility to treatments with curcumin, which reflects the importance of this natural product as a tool for the development of new epigenetic drugs which could be also explored to fight cancer in pet dogs.

## Financial support

This study was supported by grants from Research Foundation of the State of São Paulo (FAPESP - 2012/23333-2).
